# Dietary Rhythms and MASLD-Related Hepatocellular Carcinoma

**DOI:** 10.3390/cancers16203481

**Published:** 2024-10-14

**Authors:** Nadia Malakmahmoudi, Roberta Pisu, Ezio Laconi, Fabio Marongiu

**Affiliations:** Department of Biomedical Science, University of Cagliari, 09124 Cagliari, Italy; n.malekmahmoudi@studenti.unica.it (N.M.); r.pisu9@studenti.unica.it (R.P.); fabiomarongiu@unica.it (F.M.)

**Keywords:** time-restricted feeding/eating, MASLD, HCC, fasting, steatosis

## Abstract

**Simple Summary:**

Diet is a fundamental determinant of health and longevity. This relationship is complex. Historically, among the most investigated variables have been quality and quantity of food. However, over the past 2 decades increasing attention has been devoted to dietary rhythms, including time of food consumption during the day and length of fasting intervals. In this respect, contrasting evidence has recently emerged in the literature regarding the effect of adequate intervals of fasting on the evolution of diet-related chronic liver diseases, including hepatocellular carcinoma. In this review, we briefly refer to this evidence and propose a hypothesis that may help reconcile apparently conflicting observations.

**Abstract:**

Dietary rhythms have emerged as a relevant variable in the equation relating nutrition and health. Both experimental and epidemiological studies point to potential beneficial effects of adequate fasting intervals between meals on the evolution of chronic diseases associated with aging. Metabolic dysfunction-associated steatotic liver disease (MASLD) is eminently related to diet and unsurprisingly, diet-based approaches are a mainstay in countering its long-term clinical evolution, including the emergence of hepatocellular carcinoma (HCC). We briefly discuss current evidence linking fasting intervals, MASLD, and HCC and propose a working hypothesis to reconcile some of the apparently conflicting results. This hypothesis relates the beneficial effects of time-restricted eating schedules to the quantity and quality of food, and it is easily amenable to testing.

## 1. Metabolic Dysfunction-Associated Steatotic Liver Disease (MASLD) and HCC

### 1.1. From MASLD to MASH to HCC

Despite an overall decrease in cancer prevalence, the incidence of liver cancer is globally on the rise. It is the sixth most prevalent cancer worldwide and occupies the third leading position in cancer-related mortality rates [1]. This underscores the necessity of addressing the impact of recognized risk factors, including the leading role of metabolic dysfunction-associated steatotic liver disease (MASLD) [2,3,4,5].

The initial finding of an association between MASLD and HCC dates back to the 1990s [6]. Approximately one-third of the global population has MASLD, and around 20% of them progress to metabolic dysfunction-associated steatohepatitis (MASH), which can eventually lead to cirrhosis and HCC. MASLD is currently the fastest growing cause of HCC in the United States and parts of Europe, and this trend is expected to parallel the global obesity epidemic [7].

MASLD encompasses a spectrum of chronic liver conditions that originate from the accumulation of excess triglycerides in hepatocytes (steatosis), evolve through stages of liver inflammation and cellular injury (MASH), and ultimately lead to hepatic fibrosis and cirrhosis. The progression to HCC plays a pivotal role in MASLD-associated mortality [8].

The severity of MASLD is quantified according to the percentage of hepatocytes exhibiting steatosis, categorized into mild (0–33%), moderate (33–66%), and severe (>66%) [9,10]. Notably, individuals progressing to MASH display significantly elevated levels of saturated fatty acids in their triglycerides in contrast with less severe forms of fatty liver disease [11]. The increasing incidence and severity of MASLD is paralleled by a rising prevalence of obesity in the general population. There is in fact a significant association between obesity and the development of simple steatosis, as well as its progression to more advanced stages such as MASH [12]. However, it is important to point out that MASLD can also develop in individuals with a normal weight (lean/non-obese MASLD), accounting for about 20% of cases [13] (discussed in the next section).

Insulin resistance plays a pivotal role in the emergence and progression of MASLD, with diabetes mellitus acting as a key prognostic indicator for the transition from simple steatosis to more severe forms of the disease, especially towards advanced liver fibrosis [14]. Peripheral insulin resistance leads to hyperinsulinemia, which in turn stimulates hepatic lipogenesis [15]. Furthermore, insulin resistance is associated with an increased release of free fatty acids from adipose tissue, contributing to the pathophysiology of metabolic alterations [16].

In fact, insulin resistance sets off a wide array of metabolic derangements, encompassing the activation of inflammatory pathways, endoplasmic reticulum (ER) stress, and oxidative stress responses. This cascade of events is likely to play a crucial role in the carcinogenic process within the context of MASH [17].

Indeed, the link between MASLD and type 2 diabetes is possibly bidirectional, in that the presence of the former is also a risk predictor for the development of the latter [18].

Moreover, MASLD is associated with increased levels of plasma leptin, which in turn promotes the release of pro-inflammatory cytokines such as tumor necrosis factor α (TNF-α) and interleukin-6 (IL-6), thereby contributing to sterile inflammation [19,20,21].

MASLD is also linked to the reduced activity of thyroid hormones, with both MASLD and HCC demonstrating associations with hypothyroidism in humans. Additionally, the administration of thyroid hormones was reported to decrease steatosis and delay the progression of MASLD in both rodent models and human studies [22,23,24].

While the stages of steatosis and fibrosis in the liver can be potentially reversible, crossing into cirrhosis implies that the possibility of reversal significantly decreases. The onset of cirrhosis markedly increases the risk of developing portal hypertension, organ failure, and HCC, highlighting the vital importance of early detection and intervention to possibly halt or at least delay progression from MASLD to MASH [25].

### 1.2. NAFLD to HCC without Local Inflammation

As already mentioned, clearcut evidence indicates that MASLD patients can develop HCC in the absence of fibrosis/cirrhosis. It is estimated that this occurs in about half of HCC cases associated with MASLD, indicating that only a subset of individuals with MASLD advance through persistent inflammation and progressive stages of fibrosis to develop HCC [26].

The first observation was made during a single-center pathological study involving 128 patients who underwent liver resection for HCC between 1995 and 2007; this study revealed that the occurrence of HCC in livers without significant fibrosis was more frequent in patients with metabolic syndrome and MASLD (65.5%) compared to those with other liver diseases (25%) [27]. Moreover, larger tumors were observed in patients without cirrhosis. Notably, despite their larger size, HCC in the context of MASLD without cirrhosis was more likely to be well-differentiated [27].

These initial findings were then confirmed by other epidemiological data. In 2011, two Japanese studies reported that from 38% to 49% of patients with MASLD-related HCC did not present any clinical sign of cirrhosis [28]. Furthermore, among 1500 patients with HCC recruited from 2005 to 2010 by the US Veterans Administration, 194 (13%) had no signs of cirrhosis, with the main risk factors being MASLD or metabolic syndrome [29]. Similarly, an Italian study published in 2016 reported no evidence of cirrhosis in 50% of patients with MASLD-associated HCC [30]. In the largest cohort ever considered, which included 1562 verified HCC cases, 16% of all cases were associated with the presence of MASLD and one-third of the latter (83 patients) had no cirrhosis [31].

HCC in the non-cirrhotic liver is frequently identified when the tumor grows to a size that causes noticeable symptoms; it can also be incidentally detected during imaging studies or due to abnormal laboratory results.

The transition from MASLD to HCC in non-cirrhotic livers is positively correlated with the degree of fat accumulation, being more rapid in patients with grade 3 (severe) steatosis [32]. Similarly, it has been shown that better glycemic control can lower the risk of developing MASLD-related HCC in non-cirrhotic livers. In a population-based study involving 392,800 patients with MASLD, it was found that type 2 diabetes mellitus, particularly when combined with the male gender, an age over 65, and smoking habits, significantly increased the risk of HCC in a non-cirrhotic background [33].

### 1.3. Local vs. Systemic Inflammation in the Pathogenesis of MASLD-Related HCC

Several studies point to a relevant role of inflammation in bridging the link between MASLD and the increased risk of HCC [34,35]. Both systemic and locally generated inflammatory mediators have been considered, although their distinct role, if any, has been difficult to dissect [36]. In fact, while MASLD can progress to MASH, fibrosis, and cirrhosis (implying chronic liver injury and local inflammation), it is also frequently associated with other components of the metabolic syndrome, including signs of systemic inflammation [37].

**Figure 1:**
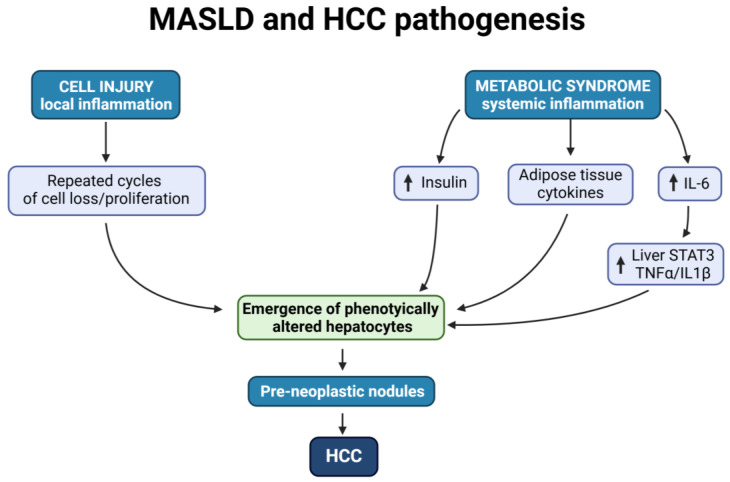
Schematic representation of pathogenetic pathways proposed for MASLD-related hepatocarcinogenesis.

The distinction between the roles of local vs. systemic inflammation becomes important in terms of disease pathogenesis (Figure 1) (see also the following paragraph).

On the one hand, there is little doubt that the local inflammatory environment associated with progressive MASLD represents a powerful biological driving force in the genesis of HCC. For example, Mittal et al. reported that the rate of progression to HCC was 10-fold higher in established MASH compared to livers with simple steatosis [29,38]. The generally agreed tenet is that chronic liver inflammation in the liver drives hepatocarcinogenesis through repeated cycles of hepatocyte injury and proliferation, thereby increasing the chances for altered putative preneoplastic cells to emerge as focal lesions or nodules/adenomas, followed by the progression to overt cancer [39,40,41]. Thus, based on studies in mice, it was proposed that injured hepatocytes release IL-1α, which in turn promotes IL-6 production, the compensatory proliferation of parenchymal cells, and hepatocarcinogenesis [40]. Similarly, the selective ablation of IL-6 expression in Kupffer cells was shown to decrease toxic liver injury, the ensuing inflammatory response and tumorigenesis in HCC-prone, Mdr2-deficient mice [42]. Furthermore, the dysregulation of neurofibromatosis-2 in experimental models of MASLD leads to the downstream activation of yes-associated protein (YAP) and its partner, TAZ, fueling liver growth and hepatobiliary carcinogenesis [43]. A role of parenchymal cell necrosis in MASLD-associated HCC was also suggested by studies based on genetic mouse models of blocked necroptosis (*Ripk3^−/−^ or Mlkl^−/−^*). It was reported that blocking necroptosis reduced markers of inflammation (including TNF-α and IL-6), monocyte infiltration, and the incidence of HCC in mice fed a choline-deficient and high-fat diet [44].

On the other hand, Park et al. were among the first to report that obesity per se, in the absence of chronic liver damage, was able to promote chemically induced HCC [35]. Both genetic- and diet-induced obesity enhanced hepatocarcinogenesis, an effect that was critically dependent on the activation of the IL-6-STAT3 pathway [35,45]. Interestingly, adipose tissue was found to be a major source of IL-6 production during systemic inflammation associated with aging [46]. It is noteworthy that brown adipocytes are also responsible for the sharp increase in the systemic levels of IL-6 following acute stress, leading to an impairment in the ability to cope with chronic ailments, including cancer [47]. Moreover, as discussed in the preceding paragraphs, between 40 and 50% of MASLD-associated HCC occur in non-cirrhotic livers, while this proportion is lower than 10% when HCC develops in a background of HCV chronic hepatitis [30], further suggesting that local inflammation may be dispensable along the pathway leading from MASLD to HCC. For example, in a series of 157 patients with HCC, arising in a background of histologically proven non-cirrhotic hepatic steatosis, 80% of the specimens were found to have no fibrosis and only a minority (15%) had signs of steatohepatitis [48].

The finding that MASLD can increase the risk of HCC in the absence of obvious chronic liver damage and local inflammation implies that alternative and/or complimentary mechanisms, other than increased cell turnover and compensatory proliferation, must be implicated as possible biological drivers of hepatocarcinogenesis under these conditions. Different possibilities have been considered. As already mentioned, an important role is thought to be played by increased systemic levels of inflammatory mediators, such as IL-6, which is frequently observed in obesity and MASLD [35]. In the liver, this results in the activation of STAT3 and overexpression of other inflammatory cytokines, including TNFα and IL1β, all of which have been linked to tumor promotion [49]. Thus, knock-out mice for IL-6 (*IL-6^−/−^*) are less susceptible, compared to the WT strain, to the enhancing effect of obesity on HCC development [35].

Hyperinsulinism related to impaired glucose metabolism and insulin resistance, which are common in individuals with MASLD [50], could also play a role in this context [51]. In fact, insulin is a prominent factor for hepatocyte cell division in itself [52] and is also able to increase the expression of insulin-like growth factor 1 (IGF-1), which in turns promotes hepatocyte proliferation [53]. Importantly, implanting pancreatic islets in the liver induces hepatocarcinogenesis in diabetic animals, and the effect has been attributed to increased intracellular insulin signaling via pathways associated with cell growth and proliferation [54].

A contribution to the development of HCC in obese individuals with non-cirrhotic hepatic steatosis could also originate from adipose tissue cytokines. More specifically, increased levels of leptin, which was shown to be mitogenic on HCC cell lines in vitro [55] and decreased levels of adiponectin, which protects against liver carcinogenesis [56], are commonly found in MASLD and obesity and may therefore fuel the development of HCC [57].

### 1.4. How Does Inflammation Promote HCC?

While the association between local and/or systemic inflammation and cancer is well established, its mechanistic basis is still being elucidated. In general, a selective effect must be exerted on mutant putative preneoplastic cells to foster their clonal expansion on the path towards neoplasia [58]. How could an inflammatory environment possibly enact such a selective effect? A clue towards illustrating this paradigm is the emergence of DNA methyltransferase 3α (Dnmt3a) mutant clones driven by IFNγ signaling in the hematopoietic tissue [59]. Using a mosaic mouse model harboring a bone marrow reconstituted with *Dnmt3a^−/−^* and *WT* HSCs, Hormaechea-Agulla et al. observed the substantial expansion of *Dnmt3a^−/−^* clones following chronic mycobacterial infection [59]. Treatment with recombinant IFNγ alone was able to induce a similar response in *Dnmt3a^−/−^* cells. It is important to note that Dnmta3a-defective clones display widespread modifications in methylation patterns, as expected, and reduced differentiation potential upon exposure to IFNγ [59]. A similar scenario has also been proposed for the TET2 loss of function in HSCs, the second most common genetic lesion found in age-associated clonal hematopoiesis [60]. TET2 mutants contribute to inducing an inflammatory environment and can better thrive in it; moreover, under these conditions, they express a block in differentiation and a proliferative advantage over WT HSCs [60]. Thus, the bone marrow stands as a prototype tissue in which inflammation provides a driving force for the selective clonal expansion of genetically altered clones, some of which can progress towards malignancy [61]. A similar paradigm was recently proposed to account for the promoting effect of air pollutants on lung carcinogenesis [62]. It was shown that exposure to environmental particulate matter causes an influx of macrophages into the lung and the subsequent release of pro-inflammatory IL1β, which in turn induces a progenitor-like phenotype within pre-existing mutant lung epithelial cells, thereby fueling their selective clonal expansion [62]. No such straightforward evidence has been gathered for the liver so far. However, it was reported that early preneoplastic lesions induced in mouse liver by neonatal exposure to diethylnitrosamine had a differential nuclear accumulation of serine 727 (S727)-phosphorylated STAT3 compared to the surrounding normal liver [63]. STAT3 phosphorylation at the S727 position is known to greatly potentiate its transcriptional activity [64], with a bias towards genes related to the hallmarks of cancer [65]. Notably, the pro-inflammatory cytokine IL-6 is a strong inducer of STAT3 activation [64], implying that local and/or systemic inflammation could induce S727 STAT3 phosphorylation in altered preneoplastic hepatocytes, driving their selective growth vis-a-vis surrounding counterparts. More recently, reduced sensitivity to the growth-suppressive effect of IFN-β was proposed as a mechanism favoring the emergence of transformed cell populations in the context of chronic hepatitis [66]. The selection of IFN-β-resistant hepatocytes was reproduced by long-term passaging in culture of a non-transformed hepatocyte line, and this phenotype was relatively stable over time [66].

## 2. MASLD, Dietary Rhythms, and HCC

### 2.1. Dietary Rhythms and MASLD

MASLD is eminently related to diet and lifestyle, and it is therefore unsurprising that several diet-based approaches have been suggested and are being explored to mitigate its impact on human health worldwide [67]. More specifically, a controlled intake of calories coupled with physical activity is considered as a standard intervention strategy to ameliorate the outcome of MASLD in a clinical setting [68,69]. For example, a green Mediterranean diet was able to reduce the prevalence of liver steatosis by over 50% in the DIRECT-PLUS 18-month randomized clinical trial [70]. Furthermore, a general positive correlation has been reported between the effectiveness of a dietary regimen in reducing body weight and in its ability to decrease the degree of steatosis and/or steatohepatitis/fibrosis [71].

In addition to the number of calories and type of macronutrients (carbohydrates vs. proteins vs. fat), increasing attention has been dedicated over the past decade to the possible impact of dietary rhythms on metabolic health and specifically on the development and progression of MASLD [72]. Several protocols alternating various fasting and feeding intervals have been proposed and tested both in humans and in experimental settings [73]. Hatory et al. were among the first to report that time-restricted feeding (TRF, consisting of 8 h of access to food followed by 16 h of fasting) with no reduction in total food intake was able to prevent chronic metabolic alterations, including liver steatosis, in mice fed a high-fat diet [74]. Similar results were later described in mice fed a “Western diet” (made of a high-fat diet of lard, milkfat, and Crisco with sugary drinking water) and exposed to TRF [75]. However, a recent study performed in rats reported no significant reduction in liver steatosis following the application of a TRF schedule in animals receiving an obesogenic diet [76]. In humans, strategies aimed at manipulating eating schedules can be grouped into two main approaches. Intermittent fasting (IF) usually includes two non-consecutive days of partial/complete fasting over a week, while time-restricted eating (TRE), like TRF protocols in experimental animals, limits food consumption to a defined time interval every day, usually 8–10 h.

A pilot study was performed on obese adult subjects, which included mostly women (41/46) and were prevalently of African American ancestry (34/46). One group (23 subjects) was invited to follow a TRE protocol for 12 weeks, with food consumption limited to 9 h per day, while the other continued to eat according to the habitual schedule and served as the control. Slight decreases in body weight (−2.6%) and systolic blood pressure (−7 mm Hg) were recorded in the TRE group at the end of 12 weeks; on the other hand, fat mass, lean mass, visceral fat mass, diastolic blood pressure, plasma LDL, and HDL cholesterol were unchanged [77]. Of note, the TRE schedule was associated with a sizeable reduction (about 20%) in total daily calorie intake. In a similar paired-sample trial conducted on 19 individuals with diagnosed metabolic syndrome, the application of the TRE regimen (eating time limited to 10 h per day) for 12 weeks led to a reduction in waist circumference, percent body fat, and visceral fat together with a lowering of blood pressure, atherogenic lipids, and glycated hemoglobin compared to pre-trial values [78]. No data were reported on hepatic lipid accumulation. Again, there was a slight (8.62% ± 14.47%) but significant (*p* < 0.007) decrease in mean daily caloric intake during the intervention period in comparison to the baseline [78]. More recently, attempts have been made to try to separate the beneficial effects of TRE from those associated with caloric restriction (CR) per se. A study by Cai et al. [72] suggested that TRE and CR can exert additive beneficial effects on body weight loss and dyslipidemia in individuals with MASLD. Participants were instructed to eat their meals within an 8 h window (TRE protocol), while the control group had no time restriction. Both groups were monitored at baseline and at 4 and 12 weeks thereafter. Energy intake was about 1350 kcal/day, consistent with a CR regimen for adult subjects, with no intergroup differences throughout the study. Body weights and fat mass decreased in both groups, but the difference from baseline was slightly (but significantly) more pronounced in subjects following the TRE protocol.

However, these findings were not confirmed by subsequent studies. Liu et al. [79] investigated 139 individuals with obesity who were invited to follow either a TRE schedule (between 8 a.m. and 4 p.m.) associated with CR or the CR regimen alone for 12 months. The CR regimen consisted of 1500 to 1800 kcal per day for men and 1200 to 1500 kcal per day for women. At the end of the observation period, a decrease in body weight was seen in both the TRE+CR and in CR-only groups (−8.0 and −6.3 kg, respectively), with no intergroup difference. In addition, significant decreases compared to baseline were recorded in other indexes related to metabolic health, including waist circumferences, BMI, body fat, body lean mass, blood pressure, and plasma parameters. The authors concluded that TRE provides no additional benefit to CR in ameliorating metabolic risk factors in obese subjects.

Using the same cohort of subjects, Wei et al. [80] measured the effect of TRE+CR vs. CR-only on intra-hepatic triglyceride content in subjects with obesity and MASLD. A total of 88 individuals (mean age 32 years) of both sexes (49 men, 39 women) were randomly invited to follow a TRE+CR (eating time between 8 a.m. and 4 p.m.) or CR-only protocol. As mentioned above, caloric intake was limited to 1500–1800 kcal for men and 1200–1500 kcal for women. After 6 months of observation, liver triglyceride content (assessed by magnetic resonance imaging) was reduced by 8.3% in the TRE+CR group and by 8.1% in the CR-only group. A similar trend was seen at 12 months, where steatosis decreased by 6.9% in individuals who followed the TRE+CR regimen and by 7.9% in the CR-only group. Liver stiffness was also significantly reduced in both groups, with no extra benefit provided by TRE schedule over the CR protocol [80].

It is clear from the above that more information is needed to fully evaluate the potential role of TRE in modulating the evolution of MASLD, alone or in combination with other lifestyle variables [81].

At least two distinct and possibly complimentary mechanisms have been proposed to explain the effect of TRE on metabolism. The first relates to the alignment of dietary rhythms to the internal diurnal metabolic clocks centrally dictated by the supra chiasmatic nucleus (SCN). The SNC entrains peripheral functional activities according to light/darkness daily cycles, thereby contributing to the establishment of circadian rhythms. However, food intake can also impact peripheral metabolic activities in several tissues, resulting in the alignment or misalignment with SCN signaling. When food intake is aligned with the central clock, the amplitude of circadian metabolic oscillations increases, while a misalignment will result in the opposite effect. A progressive reduction in the amplitude of circadian metabolic oscillations is observed with aging [82], while their reinforcement, as with aligned TRE strategies, helps in preserving metabolic health [83]. In addition, TRE exerts profound effects on the regulation of metabolic pathways involved in energy balance, nutrient signaling, mitochondrial efficiency, proteostasis, and autophagy [84], all of which have been implicated in sustaining healthy aging [84,85].

### 2.2. Dietary Rhythms and HCC

In 1994, a study by Grasl-Kraupp et al. proposed that fasting could antagonize early phases of liver carcinogenesis by inducing the clearance of preneoplastic cells through apoptosis [86]. However, a few years later, results from our research group indicated that long periods of fasting (cycles of three consecutive days) could in fact favor the growth of preneoplastic nodules and foster their progression to overt neoplasia in a well-characterized experimental model [87]. This suggested that the effect of fasting could be different (and in fact, opposite) depending on the duration and the specific experimental context. More recently, a protocol of rather drastic TRF (only 2 h of access to food every day), applied for 16 weeks, was reported to reduce the progression to cirrhosis and HCC in rats chronically exposed to the carcinogen diethyl-nitrosamine [88]. Of note, such an extreme TRF protocol also implied a 30 to 40% decrease in food intake compared to ad libitum-fed controls, making it difficult to discern the effect of TRF per se from that of the associated CR. A less stringent TRF schedule was used in a subsequent study. Mice spontaneously developing HCC were assigned to a dietary regimen based on a “Western” diet consisting of 8 h of food availability per day, with a follow up of 28 weeks. At the end of the study, the incidence and the size of the neoplastic lesions in the liver were sharply decreased in the TRF group compared to controls. TRF also reduced hepatic steatosis, inflammation, and the fibrosis score. Importantly, these effects were observed in the absence of significant differences in food consumption between TRF and control groups and were therefore entirely assigned to the change in eating patterns [89]. Using a different experimental setting, TRF was reported to delay the emergence of what is referred to as the age-associated neoplastic-prone tissue landscape in rat livers [90]. The growth of transplanted preneoplastic hepatocytes, which is enhanced in aged livers [91], was found to be reduced when animals, fed a regular chow diet, were exposed to a TRF regimen throughout life [90].

In humans, a very recent publication reporting on the National Health and Nutrition Examination Survey (NHANES) conducted on the civilian noninstitutionalized population in the United States [92] found an inverse correlation in both elderly men and women between the duration of overnight fasting time and mortality from any cancer. After stratifying individuals into four quartiles according to the duration of overnight fasting (Q1: ≤7.5 h, Q2: >7.5 h–≤10.58 h, Q3: >10.58 h–≤12.38 h, Q4: >12.38 h) and using the Q3 group as the cohort of reference, the hazard ratios for dying from cancer were 0.87, 1.35, and 1.41 for the Q4, Q2, and Q1 cohorts, respectively. However, overall mortality did not follow a similar trend and was the lowest for the Q2 and Q3 cohorts, increasing in both people with the shortest (Q1) and in those with the longest (Q4) overnight fasting interval. No direct information is yet available on any effect of TRE, specifically on the development and/or progression of human hepatocarcinoma; however, dietary interventions exerting a beneficial effect on MASLD progression were also found to be associated with a decrease in the incidence of and mortality from cancer, including HCC [93].

While it is premature at this point to speculate on any possible mechanism mediating the effect of TRE/TRF on hepatocarcinogenesis, it is reasonable to predict that the improvement of metabolic health might be a key component. For example, in a mouse model of chemically induced lung carcinogenesis, TRF was found to delay neoplastic development, and the effect was associated with a reshaping of circadian metabolism and the upregulation of autophagy [94].

## 3. Conclusions

**Figure 2:**
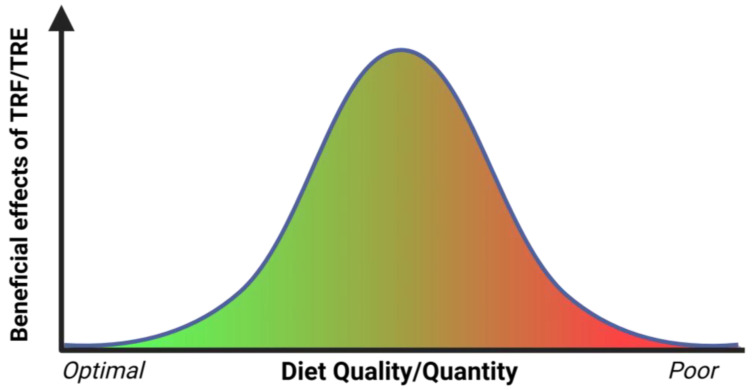
Hypothesis proposing a non-linear relationship between quality/quantity of diet and the effect of time-restricted feeding/eating.

MASLD is a major health problem worldwide. As it is implicit in its updated definition (an evolution from the original non-alcoholic fatty liver disease, NAFLD), chronic dietary-induced metabolic modifications are at the heart of the pathogenesis of MASLD, which can evolve into fibrosis, cirrhosis, and HCC. Very recently, a study reporting on a phase 3 randomized controlled trial has suggested that resmetirom, an oral liver-directed thyroid hormone receptor beta-selective agonist, could improve (delay) the evolution of liver fibrosis in individuals with non-alcoholic steatohepatitis [95]. Although these results are of interest, a mainstay in the management of MASLD remains based on dietary and lifestyle interventions, i.e., strategies that attempt to tackle the very root of the disease process [96]. While the quantity and quality of nutrients are central to these interventional strategies, a complimentary component that has emerged in the dietary equation pertains to the eating schedule. Studies in animal models, carried out mostly in mice, have in fact supported the concept that adequate intervals of fasting, distributed on a daily or weekly basis, can help in restoring metabolic health during the evolution of MASLD. These concepts have also been extended and tested in humans [73]. Moreover, it is pertinent to note that drug candidates that are being investigated for the treatment of MASH are specific for metabolic pathways that are under circadian control [97], providing support to the argument that dietary rhythms play a role in disease pathogenesis. However, results from clinical trials are far from being conclusive and have in fact led to conflicting results, illustrating the need for more investigations using carefully designed protocols [81]. For example, a recent investigation on mice fed a MASLD-inducing, choline-deficient, and high-fat diet, failed to observe any beneficial effect of a TRF schedule over ad libitum feeding on the evolution of liver pathology [98]. Based on the available evidence, we propose the existence of a non-linear relationship in the effects of dietary protocols involving daily fasting (TRF/TRE) and metabolic health (Figure 2).

According to this hypothesis, the impact of TRF/TRE strategies is critically dependent on diet composition, including both the quantity and quality of nutrients. If the diet composition is optimal, any beneficial effect of TRF/TRE tends to be negligible under conditions of pre-existing good metabolic health. At the other end of the spectrum, if the dietary composition is poor, any beneficial effect of TRF/TRE is probably overrun by the overwhelming negative consequences of such a diet. If, however, diet composition is sub-optimal but not too poor, then a TRF/TRE approach can express its beneficial impact at its best. While experimental studies tend to focus on extreme conditions by design, real-world human experience is possibly more of the sub-optimal/not-too-poor type, making it more likely to be amenable to the beneficial effects of TRE. We acknowledge the fact that defining what is the optimum quantity and even more so, the optimum quality of a diet, is not an easy task. Nevertheless, trying to identify the best conditions under which strategies based on chrono-nutrition can exert their potential benefits is a most exciting challenge facing research in this field.
